# Asthma and Cacosmia Could Be Predictive Factors of Olfactory Dysfunction Persistence 9 Months after SARS-CoV-2 Infection: The ANOSVID Study

**DOI:** 10.3390/life12070929

**Published:** 2022-06-21

**Authors:** Can Tipirdamaz, Souheil Zayet, Molka Osman, Julien Mercier, Elodie Bouvier, Vincent Gendrin, Kévin Bouiller, Quentin Lepiller, Lynda Toko, Alix Pierron, Pierre-Yves Royer, Pauline Garnier, N’dri-Juliette Kadiane-Oussou, Catherine Chirouze, Timothée Klopfenstein

**Affiliations:** 1 Infectious Disease Department, Nord Franche-Comté Hospital, 90400 Trevenans, France; can.tipirdamaz@gmail.com (C.T.); ukaliq.eb@gmail.com (J.M.); vincent.gendrin@hnfc.fr (V.G.); lynda.toko@hnfc.fr (L.T.); alix.pierron@hnfc.fr (A.P.); pierre-yves.royer@hnfc.fr (P.-Y.R.); kadianeoussou14@gmail.com (N.-J.K.-O.); timothee.klopfenstein@hnfc.fr (T.K.); 2 Faculty of Medicine of Tunis, University Tunis El Manar, Tunis 1007, Tunisia; molkaosman@gmail.com; 3 Clinical Research Unit, Nord Franche-Comté Hospital, 90400 Trevenans, France; elodie.bouvier@hnfc.fr; 4 Infectious Disease Department, University Hospital of Besançon, 25000 Besançon, France; kbouiller@chu-besancon.fr (K.B.); catherine.chirouze@univ-fcomte.fr (C.C.); 5 Virology Department, University Hospital of Besançon, 25000 Besançon, France; q1lepiller@chu-besancon.fr; 6 Microbiology Department, Nord Franche-Comté Hospital, 90400 Trevenans, France; pauline.garnier@hnfc.fr

**Keywords:** COVID-19, olfactory dysfunction, cacosmia, asthma, severity

## Abstract

**Background.** Long-term evolution data of olfactory disorders (OD) in COVID-19 are limited. **Method.** ANOSVID is a retrospective study in Nord Franche-Comté Hospital (France) that included COVID-19 patients from the first wave. The aim was to describe OD evolution, especially in patients with persistent OD (p-OD group) in comparison with patients with resolved OD (r-OD group). **Results.** Among 354 COVID-19 patients, 229 reported OD were included. Eighty-five percent of patients (*n* = 195) recovered from their OD within 90 days. However, 9.5 months (in average) after symptoms onset, OD were persisting in 93 patients (40.6%) and resolved in 136 patients (59.4%). In the p-OD group (*n* = 93), the mean age was 51.4 years (19–98) ± 20.2, and 65 patients (69.9%) were female; the three main comorbidities in the p-OD group were: asthma (20.4%, *n* = 19), allergic rhinitis (19.4%, *n* = 18), and arterial hypertension (16.1%, *n* = 15). Eleven patients (12%) presented anosmia, and 82 patients (88%) presented hyposmia. Asthma was more described in p-OD group than r-OD group (19 (20.4%) versus 10 (7.4%), *p* = 0.006). Cacosmia was more described in p-OD group than r-OD group (27 (29.0%) versus 18 (13.2%), *p* = 0.005). There was no significant difference between the two groups concerning other comorbidities and symptoms, clinical, biological, and imaging findings, and outcome or about the impact of OD on the quality of life of the patients between the p-OD group and r-OD group. sQOD-NS brief version score was 10.7 ± 5.89 and 12.0 ± 6.03, respectively (*p* = 0.137). **Conclusion.** Forty-one percent of patients with OD reported OD persistence 9.5 months after COVID-19 (hyposmia in 88% of cases). Asthma and cacosmia could be predictive factors of OD persistence.

## 1. Introduction

Coronavirus disease 2019 COVID-19 is a viral emerging infectious disease caused by the severe acute respiratory syndrome coronavirus 2 (SARS-CoV-2). In the first clinical studies from Eastern Asia, the most common symptoms described in COVID-19 were mainly general, respiratory, and gastro-intestinal symptoms [1]. Nevertheless, after the spread of the disease in Europe, additional studies highlighted new common symptoms, such as olfactory and gustatory dysfunctions [2]. These olfactory disorders (OD) are defined as partial (hyposmia) or complete (anosmia) loss of smell. Studies demonstrated that anosmia could be a specific symptom of COVID-19, especially when associated with dysgeusia, which is helpful to contribute to early diagnosis [3,4]. Several studies reported that COVID-19 patients with OD were more frequently described in young female with fewer comorbidities (especially cardiovascular) [2,5].

Pathogenesis data from clinical studies suggested that these symptoms are related to a neurological disorder, but the exact mechanisms associated with COVID-19 anosmia remain unclear [6,7]. According to some authors, anosmia is due to inflammation in the olfactory epithelium [8]. However study in animal models shows that coronavirus can transneuronally disseminate into the brain through neuro-olfactory pathway after having invaded the olfactory neuroepithelium integrity as a consequence of disruption of the olfactory neuroepithelium [6].

Indeed, the duration of OD is still unclear. Several studies reported a quick recovery in 7–10 days in the majority of patients [2,9], while others reported persisting symptoms even several months after the onset of the disease [10,11,12,13]. These persistent symptoms could be included in a new nosological entity that some authors referred to as “long-COVID-19 syndrome” [14,15] or “post-COVID-19 syndrome” [16,17], describing people who have symptoms for more than 28 days after the onset of the disease [14].

On 1 March 2020, the first major French cluster of COVID-19 began in the city of Mulhouse (less than 30 miles from our hospital) [18]. At this time, we conducted a first study with the 54 first patients, which revealed that 47% of them presented OD with a favorable outcome usually in less than 28 days [18]. The aim of this study is to describe persisting OD in patients with confirmed SARS-CoV-2 infection.

## 2. Materials and Methods

### 2.1. Study Population

ANOSVID was an observational retrospective study in *Nord Franche-Comté* Hospital (HNFC), France, with methodological details as recently described [11]. We included all adult (≥18 years old) with COVID-19 confirmed from 1 March 2020 to 31 May 2020. We excluded from this study any patient declining to participate in the study or who did not respond the online questionnaire.

We present here the results of patients with persistent OD (anosmia or hyposmia). The primary outcome was to describe demographic characteristics; comorbidities; biological, virological, and radiological findings; and the symptoms of patients with persistent OD. The secondary outcome was to compare patients with persistent OD (p-OD group) and patients with resolved OD (r-OD group).

### 2.2. Clinical and Paraclinical Data

Clinical data were collected through an online questionnaire. Concerning the quality of life related to anosmia, we used the Brief version of the QOD-NS [19]. Based on Leclercq et al. [20], the French QOD-NS is a reliable and valid self-administered tool in the evaluation of the impact of OD on quality of life of French-speaking patients.

In case of hospitalization, its characteristics (duration, intensive care unit admission (ICU), outcome, and treatment) were collected through the medical records such as biological, virological, and radiological findings.

Patients with resolved OD were defined by patients who recovered their olfactory function as it was before SARS-CoV-2 infection. The persistence of olfactory dysfunction (p-OD) was defined by the presence (at the date when the questionnaire was answered) of olfactory dysfunction related to SARS-CoV-2 infection and which was not present before SARS-CoV-2 infection. In the p-OD group, patients who related a final date for OD due to SARS-CoV-2 infection before relapse were considered to have a “recurrence of OD” after an asymptomatic period. The patients who had a persistence of OD since the beginning of SARS-CoV-2 infection (without an asymptomatic period) were considered to have “continuous OD”.

### 2.3. Statistical Analysis

Continuous variables were expressed as mean and standard deviation (SD) and compared with Student’s *t*-test. Categorical variables were expressed as number and percentage (%) and compared by chi-square test or Fisher’s exact test between the two groups (patients with persistent OD and patients with resolved OD). A *p*-value < 0.05 was considered significant. We used the R++ v1.4.02 software (ZEBRYS, Toulouse, France).

## 3. Results

During the study period, 354 COVID-19 patients were included in our facility out of 1138 [21]. Among these patients, 229 presented OD and were included in the study. Nine and half months after the onset of the first symptoms, OD were resolved in 136 patients (r-OD group, 59.4%) and were persisting in 93 patients (p-OD group, 40.6%) (Table 1).

### 3.1. OD Characteristics after SARS-CoV-2 Infection

**Figure 1:**
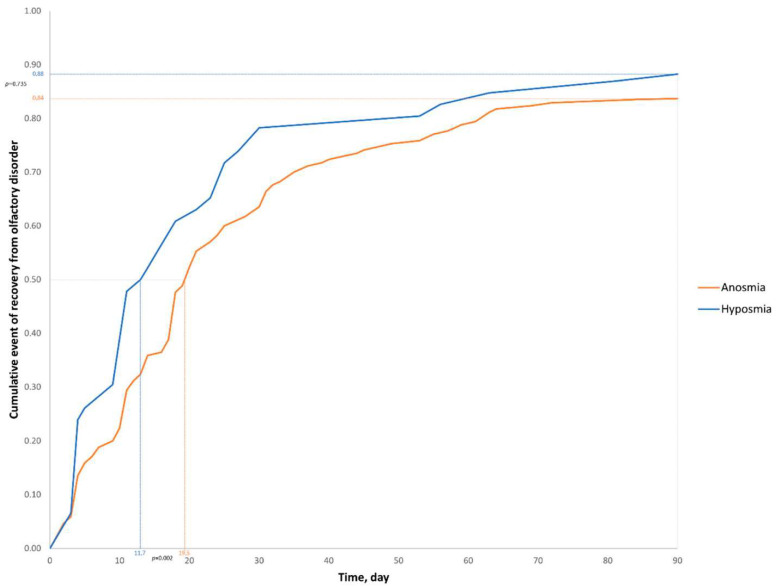
Olfactory recovery over tie in the anosmia (*n* = 180) and hyposmia (*n* = 49) groups after SARS-CoV-2 infection.

From these 229 patients with OD, 180 patients (78.6%) had anosmia, and 49 (21.4%) had hyposmia. They answered the questionnaire with a mean of 283.9 days (a mean of 9.5 months) ± 26.1 (211–366) after symptoms onset. Olfactory recovery was significantly faster for patients with hyposmia than patients with anosmia. The median of olfactory recovery was 11.7 days for patients with hyposmia and 19.5 days for patients with anosmia (*p* = 0.002). At day 90, there was no significant difference in the olfactory recovery rate between patients with hyposmia and patients with anosmia (respectively, 88.3% and 83.7%, *p* = 0.735) (Figure 1). One hundred ninety-two of patients with OD (83.4%) had gustatory disorder associated (Table 2). The impact of OD on the quality of life of the patients is reported in Table 3. Patients particularly complained about the loss of appetite; the mean rate of this item was 0.97 ± 1.13 (from a scale of 0–3, with higher score reflecting better olfactory-specific quality of life).

### 3.2. Description of Patients with Persistent OD after SARS-CoV-2 Infection

**Figure 2:**
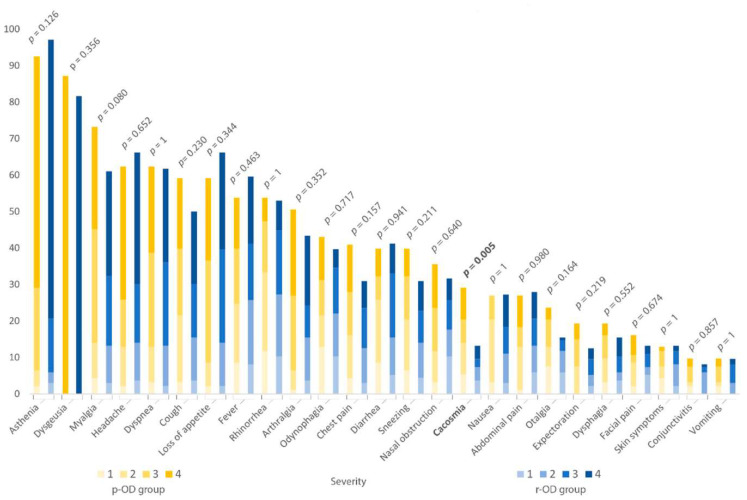
Proportion of symptoms in the OD-group (*n* = 93) and in the r-OD group (*n* = 136) at the onset of the disease.

**Figure 3:**
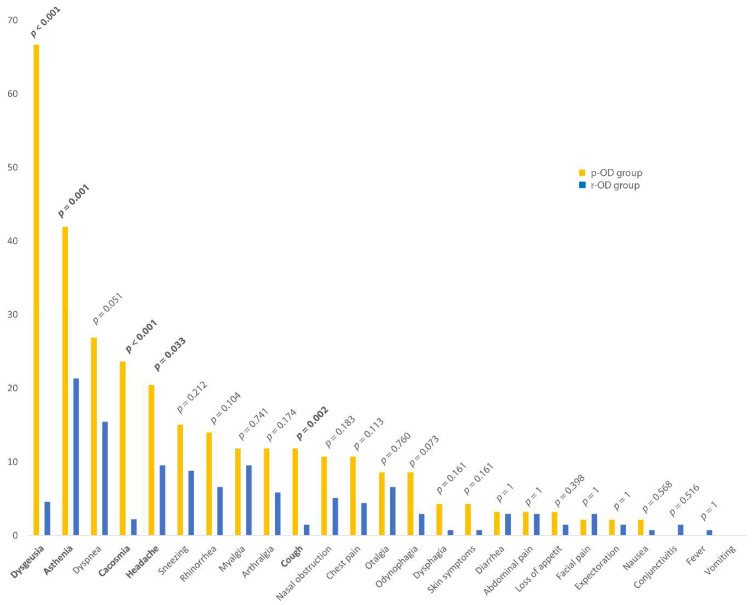
Proportion of persistent symptoms in the OD-group (*n* = 93) and in the r-OD group (*n* = 136) after SARS-CoV-2 infection.

Among the 93 patients in the p-OD group, 11 patients (12%) presented anosmia, and 82 patients (88%) had only hyposmia; 75 patients (80.6%) reported a recurrence of OD after recovery, and only 18 patients (19.4%) patients reported continuous OD (persistence of OD since the beginning of SARS-CoV-2 infection without an asymptomatic period). In the p-OD group, the mean age of patients was 51.4 years ±20.2, and 65 were female (69.9%). Fifty-six patients (60.2%) had underlying comorbidities. The three main comorbidities were: asthma (20.4%, *n* = 19), allergic rhinitis (19.4%, *n* = 18), and arterial hypertension (16.1%, *n* = 15). More than one-third of these patients were hospitalized (37.6%, *n* = 35), and five patients (14.3%) were transferred to ICU. Concerning biological data in hospitalized patients, the mean C-reactive protein was 117.3 mg/L ±86.8. Twenty-seven patients had thoracic computed tomography, and all of them presented ground-glass opacities (GGO). (Table 1). The most common symptoms on the onset of COVID-19 associated to OD were asthenia (92.5%, *n* = 86), dysgeusia (87.1%, *n* = 81), myalgia (73.1%, *n* = 68), headache (62.4%, *n* = 58), and dyspnea (62.4%, *n* = 58). Cacosmia was also present in 27 patients (29%) (Figure 2). The most common persisting symptoms associated with OD were dysgeusia (66.7%, *n* = 54), asthenia (41.9%, *n* = 39), dyspnea (26.9%, *n* = 25), and cacosmia (23.7%, *n* = 22) (Figure 3).

### 3.3. Comparison of Two Groups

The mean age was 51.4 ± 20.2 years in p-OD group and 49.6 ± 18.1 years in r-OD group (*p* = 0.505), without sex predominance. No significant differences were found about BMI, current smoking, and in pregnant and HCWs population. Asthma was more described in p-OD group than in r-OD group (19 (20.4%) versus 10 (7.4%), *p* = 0.006). Moreover, patients in p-OD group were significantly being more treated for malignancy than r-OD group (4 (4.3%) versus 0, *p* = 0.025). Otherwise, there was no significant difference about neurological diseases rate between p-OD group and r-OD group (9 (9.7%) versus 6 (4.4%), *p* = 0.188) and other comorbidities rates. There were also no significant differences between the two groups concerning clinical, biological, and imaging findings and outcome. We found a trend for creatinine to be lower in p-OD group than r-OD group (respectively, 70.7 µmol/L ±21.6 versus 88.2 µmol/L ±57.0, *p* = 0.055); in the same way, creatinine clearance also followed this trend inversely (92.1 mL/min/1.73 m^2^ ±22.2 for p-OD group versus 83.3 mL/min/1.73 m^2^ ±22.4 for r-OD group, *p* = 0.078) (Table 1). Gustatory dysfunction prevalence and its recovery duration were not significantly different between the two groups. However, it was significantly more often persisting in p-OD group than r-OD group (53 (66.7%) versus 5 (4.6%), *p* < 0.001) (Table 2). There were no significant differences between symptoms present at the onset of the disease except for cacosmia, which was described more in p-OD group than r-OD group (respectively, 27 (29.0%) versus 18 (13.2%), *p* = 0.005) (Figure 2). Among the other persistent symptoms, four were significantly more often present in p-OD group than r-OD group: asthenia (respectively, 39 (41.9%) versus 29 (21.3%), *p* = 0.001), cacosmia (respectively, 22 (23.7%) versus 3 (2.2%), *p* < 0.001), headache (respectively, 19 (20.4%) versus 13 (9.6%), *p* = 0.033), and cough (respectively, 11 (11.8%) versus 2 (1.5%), *p* = 0.002) (Figure 3). There was no significant difference about the impact of OD on the patients’ quality of life between the two groups. Indeed, the total score of the short version of Questionnaire of Olfactory Disorders-Negative Statements was, respectively, 10.7 ± 5.89 in the p-OD group and 12.0 ± 6.03 in the r-OD group (from a scale of 0–21, with higher score reflecting better olfactory-specific quality of life) (Table 3).

## 4. Discussion

### 4.1. OD Characteristics after SARS-CoV-2 Infection

In our study, OD was present in 64.7% of the confirmed COVID-19 patients with positive RT-PCR. This result shows a higher percentage of patients presenting OD following SARS-CoV-2 infection in comparison with the systematic reviews published by Wu et al. and Tong et al., who reported 52.7% to 53.6% of OD [22,23]. This difference may be due to selection bias, as patients with OD could possibly respond more likely to the questionnaire due to the name of the study (ANOSVID). However, our results are similar to several European studies [24,25,26,27,28], especially Riestra-Ayora et al., where OD prevalence corresponds to 64.1% of the COVID-19 patients [25].

### 4.2. Patients with Persistent OD after SARS-CoV-2 Infection

In this cohort of 229 patients from the first wave of SARS-CoV-2 infection, 195 patients (85.2%) recovered their olfactory function after acute SARS-CoV-2 at day 90. However, 93 patients (40.6%) reported an OD nine and a half months after SARS-CoV-2 infection, explained by a recurrence of OD after recovery. Continuous OD seems to be uncommon (7.9%, *n* = 18).

When analyzing the p-OD group in 93 patients with persistent OD, 60.2% of patients had underlying comorbidities, and the three main comorbidities were asthma, allergic rhinitis, and arterial hypertension. Except for arterial hypertension, these results did not correspond to comorbidities usually described in COVID-19 [29]. This may be explained in part by the fact that allergic rhinitis is not frequently requested. However, these results are consistent with studies, which focus more particularly on otorhinolaryngological comorbidities as Lechien et al. one [2]. This raises the question that local inflammation in the airway epithelium could be involved in persistent OD. The most common persisting symptoms in addition to OD were dysgeusia, asthenia, dyspnea, and headache, which are consistent with the literature (thus far) [30,31,32,33].

### 4.3. Comparison of Two Groups

Our results showed that asthma was significatively more present in the p-OD group than in r-OD group, and cacosmia was more frequently described at the onset of the disease in p-OD group than in r-OD group. These two elements could be predictive factors of OD persistence after SARS-CoV-2 infection.

Sun et al. [34] suggested that Interleukine-4 (IL-4) cytokine could have a role in post-COVID-19 syndrome. IL-4 continued to significantly increase in COVID-19 patients even several months after the onset of the disease. Its elevation signals an ongoing neuroinflammation after SARS-CoV-2 infection that may influence neurological sequelae, such as OD [34]. In our results, asthma was more described in p-OD group than r-OD group. According to Afshari et al. and Lee et al.’s studies [35,36], serum IL-4 level is considerably higher in asthmatic than non-asthmatic patients. Therefore, we can hypothesize that asthma is involved in the persistence of OD because of higher levels of circulating IL-4.

Concerning biological findings, despite the low number of patients with biological findings (*n* = 84), there was a trend for creatinine level to be lower in p-OD group than in r-OD group; this trend was also found inversely for creatinine clearance. ACE2 is highly expressed in kidneys, particularly in the proximal tubules, and it is an important homeostatic component of the renin angiotensin system. Its action causes an elevation of the glomerular filtration rate (GFR) by degrading vasoconstrictor peptide angiotensin II and generating vasodilator peptide angiotensin 1–7 [37]. Suggesting kidney ACE2 gene expression was correlated to olfactory epithelium ACE2 gene expression, this might explain the observance of higher GFR in p-OD group than r-OD group.

Regarding our study, these OD were strongly associated with gustatory dysfunction. This result is consistent with literature data [2,22,23,25,27,38]. In our study, when OD were persisting, gustatory dysfunction was persisting as well in two-thirds of our population. Cooper et al. [39] demonstrated as well that some taste receptor cells could express ACE2, constituting a gateway for SARS-CoV-2 to the central nervous system. Hence, patients with both olfactory and gustatory dysfunctions that persisted should have higher ACE2 expression in both olfactory epithelium and taste receptor cells than other patients.

The ACE2 receptor is now known to be the cellular receptor for SARS-CoV-2 [40]. Many tissues express ACE2, with a prominent expression in the olfactory epithelium [41]. It is supported by the absence of ACE2 expression in neurosensory cells and the short recovery time of most ODs (7 to 10 days) excluding axonal destruction. Concomitant nasal obstruction in SARS-CoV-infection may sometime explain transmission hyposmia in some cases, as in other otorhinolaryngological infections. However, persistent anosmia raises questions; thus, a neurotropism of SARS-CoV-2 has been suggested [42], with brain imaging abnormalities found after SARS-CoV-2 infection, particularly in regions involved in smell and taste [43].

One limitation of our study is selection bias of our study population. There is a high number of HCWs, which can be explained by the restriction access of RT-PCR tests in the beginning of the first wave. We also excluded the more severe patients because patients who died where not included (cannot respond to the questionnaire). Concerning the main comorbidities (asthma, allergic rhinitis, and arterial hypertension), they were just observed; this does not mean that people with this comorbidity will develop p-OD or, further, that these data are not statistically significant except for asthma. Concerning our follow-up, it was only based on a questionnaire, which is an issue of subjectivity. Moreover we did not include new variants of interest of SARS-CoV-2, which may cause different clinical expressions such as severity [44,45] or OD expression, as suggested by Butowt et al. [46]. Use of therapeutics in our study, such as hydroxychloroquine, is also another limitation, as they no longer appeared in the standard of care treatment of COVID-19.

## 5. Conclusions

In our study, we observed that 85% of patients recovered from their OD within 90 days. However, 9.5 months (in average) after symptoms onset, OD persisted in 41% of cases as explained by a recurrence of OD after recovery. Patients with persistent OD had unusually anosmia (12%) but presented hyposmia in 88% of cases.

We identified that asthma and cacosmia at the onset of the disease could be predictive factors of OD persistence after SARS-CoV-2 infection. This might be explained by the cytopathogenesis of the coronavirus through its effective receptor, ACE2, which can be highly expressed in the olfactory epithelium.

## Figures and Tables

**Table 1:** Demographic, comorbidities, laboratory, and imaging findings in 229 COVID-19 patients with resolved or persistent olfactory dysfunction after infection with SARS-CoV-2, *Nord Franche-Comte* Hospital, *France*.

Variables	Proportion of Patients			*p*-Value
	Resolved Olfactory Dysfunction (*n* = 136) (59.4%)	Persistent Olfactory Dysfunction (*n* = 93) (40.6%)	Total (*n* = 229) (100%)	
Time between questionnaire answer and symptoms onset, day (mean, extremes, SD)	285.0 [211–335] ± 23.9	282.2 [281–366] ± 29.2	283.9 [211–366] ± 26.1	0.435
*Demographic and baseline characteristics*				
Age, year (mean, extremes, SD)	49.6 [20–94] ± 18.1	51.4 [19–98] ± 20.2	50.4 [19–98] ± 18.9	0.505
Sex, % (no.)				0.094
Male	41.9 (57)	30.1 (28)	37.1 (85)	
Female	58.1 (79)	69.9 (65)	62.9 (144)	
HCW, % (no.)	49.3 (67)	49.5 (46)	49.3 (113)	1
BMI (kg/m^2^) (mean, extremes, SD)	26.8 [17.5–43.2] ± 5.6	26.3 [15.9–47] ± 5.7	26.6 [15.9–47] ± 5.7	0.594
<18.5	3.0 (4)	8.6 (8)	5.2 (12)	0.194
[18.5–25]	40.4 (55)	35.5 (33)	38.4 (88)	0.548
[25–30]	29.4 (40)	32.3 (30)	30.6 (70)	0.741
≥30	25.7 (35)	22.6 (21)	24.5 (56)	0.708
Pregnancy, % (no.)	0.7 (1)	2.2 (2)	1.3 (3)	0.567
Current smoking, % (no.)	6.6 (9)	8.6 (8)	7.4 (17)	1
*Comorbidities*				
Comorbidities, % (no.)				
Yes	58.8 (80)	60.2 (56)	59.4 (136)	1
No	41.2 (56)	39.8 (37)	40.6 (93)	1
Arterial hypertension, % (no.)	22.8 (31)	16.1 (15)	20.1 (46)	0.290
Cardio-vascular diseases, % (no.)	30.1 (41)	23.7 (22)	27.5 (63)	0.674
Cardiac arrhythmia	4.4 (6)	7.5 (7)	5.7 (13)	0.465
Heart failure	3.7 (5)	3.2 (3)	3.5 (8)	1
Coronary heart disease	2.9 (4)	1.1 (1)	2.2 (5)	0.651
Diabetes mellitus, % (no.)	4.4 (6)	10.8 (10)	7.0 (16)	0.111
Chronic kidney failure, % (no.)	3.7 (5)	1.1 (1)	2.6 (6)	0.405
Neurologic diseases ^1^, % (no.)	4.4 (6)	9.7 (9)	6.6 (15)	0.188
ORL diseases, % (no.)	18.4 (25)	25.8 (24)	21.4 (49)	0.221
Rhinosinusitis nasal polyps	0.0 (0)	3.2 (3)	1.3 (3)	0.064
Surgical rhinoplasty	2.2 (3)	3.2 (3)	2.6 (6)	0.687
Allergic rhinitis	14.0 (19)	19.4 (18)	16.2 (37)	0.347
Chronic rhinosinusitis	3.7 (5)	5.4 (5)	4.4 (10)	0.530
**Respiratory diseases, % (no.)**	**10.3 (14)**	**23.7 (22)**	**15.7 (36)**	**0.010**
COPD	2.2 (3)	1.1 (1)	1.7 (4)	0.650
**Asthma**	**7.4 (10)**	**20.4 (19)**	**12.7 (29)**	**0.006**
Others ^2^	0.7 (1)	2.2 (2)	1.3 (3)	0.567
Past history of malignancy, % (no.)	6.6 (9)	7.5 (7)	7.0 (16)	0.982
**Actually treated**	**0.0 (0)**	**4.3 (4)**	**1.7 (4)**	**0.025**
Use of immunosuppressants,% (no.)	0.0 (0)	2.2 (2)	2.2 (2)	0.162
Psychiatric disorders, % (no.)	5.1 (7)	7.5 (7)	6.1 (14)	0.643
Depressive disorder	4.4 (6)	6.5 (6)	5.2 (12)	0.553
Others ^3^	0.7 (1)	1.1 (1)	0.9 (2)	1
*Hospitalization*				
Hospitalization, % (no.)	37.5 (51)	37.6 (35)	37.8 (86)	1
Duration of hospitalization, days (mean, extremes, SD)	12.2 [1–55] ± 12.1	12.4 [1–73] ± 14.3	12.3 [1–73] ± 13.0	0.938
*Treatments received, % (no.)*	*Over 51 hospitalized patients*	*Over 35 hospitalized patients*	*Over 86 hospitalized patients*	
Antibiotics	82.4 (42)	82.3 (29)	82.6 (71)	1
Hydroxychloroquine	66.7 (34)	71.4 (25)	68.6 (59)	0.817
Lopinavir/Ritonavir	3.9 (2)	5.7 (2)	4.7 (4)	1
Steroids (Dexamethasone)	19.6 (10)	14.3 (5)	17.4 (15)	0.727
Anti-IL-6 (Tocilizumab)	5.9 (3)	2.9 (1)	4.7 (4)	0.643
*Laboratory data*	*Over 49 analyzed patients*	*Over 35 analyzed patients*	*Over 84 analyzed patients*	
Laboratory data on admission (mean, extremes, DS)				
White-cell count/mm^3^ (4.00–10.00/mm^3^)	7.45 [2.66–23.95] ± 3.75	7.55 [3.!98–17.51] ± 3.37	7.49 [2.66–23.95] ± 3.58	0.895
Lymphocytes/mm^3^ (1.50–4.00/mm^3^)	0.95 [0.15–2.70] ± 0.50	0.95 [0.33–1.83] ± 0.42	0.95 [0.15–2.70] ± 0.46	0.991
Hemoglobin, g/dL (13.5–17.5 g/dL)	13.7 [10.4–18.2] ± 1.4	13.6 [10.5–17.0] ± 1.7	13.6 [10.4–18.2] ± 1.5	0.695
Creatinine, μmol/L (65–120 μmol/L)	88.2 [46–403] ± 57.0	70.7 [43–139] ± 21.6	80.9 [43–403] ± 44.3	0.055
Creatinine clearance CKD-EPI, mL/min/1.73 m^2^ (69–119 mL/min/1.73 m^2^)	83.3 [8–122] ± 22.4	92.1 [29–132] ± 22.2	87.0 [8–132] ± 46.3	0.078
Alanine aminotransferase, U/L (8–45 U/L)	49.6 [13–188] ± 39.2	54.0 [12–175] ± 42.5	51.4 [12–188] ± 40.1	0.660
Aspartate aminotransferase, U/L (10–40 U/L)	52.2 [11–159] ± 34.0	53.9 [17–193] ± 42.8	52.9 [11–193] ± 37.3	0.862
C-reactive protein, mg/L (<5 mg/L)	117.5 [10.1–478.7] ± 91.7	117.3 [0.1–377.6] ± 86.8	117.4 [0.1–478.7] ± 89.1	0.992
C-reactive protein >100 mg/L, % (no.)	44.9 (22)	51.4 (18)	47.6 (40)	0.712
RT-PCR SARS-CoV-2 CT E (mean, extremes, SD)	27.0 [16.4–37.6] ± 5.81	26.3 [19.6–32.5] ± 5.06	26.8 [16.4–37.6] ± 5.53	0.661
*Radiological data*	*Over 38 CT-scanned patients*	*Over 27 CT-scanned patients*	*Over 65 CT-scanned patients*	
Thoracic imaging features, % (no.)				
GGO	89.5 (34)	100 (27)	93.4 (61)	0.135
Consolidation opacities	73.7 (28)	81.5 (22)	76.9 (50)	0.662
Crazy-paving sign	44.7 (17)	44.4 (12)	44.6 (29)	1
<25% extension	55.3 (21)	59.3 (16)	56.9 (37)	0.947
>50% extension	10.5 (4)	14.9 (4)	12.3 (8)	0.709
*Complications % (no.)*	*Over 51 hospitalized patients*	*Over 35 hospitalized patients*	*Over 86 hospitalized patients*	
SARS	11.8 (6)	14.3 (5)	12.8 (11)	0.752
Transferred to ICU	15.7 (8)	14.3 (5)	15.1 (13)	1
Mechanical ventilation	15.7 (8)	14.3 (5)	15.1 (13)	1
Pleural Effusion	11.8 (6)	5.7 (2)	9.3 (8)	0.464
Hepatitis	15.7 (8)	14.3 (5)	15.1 (13)	1

**Bold: significant difference (*p* < 0.05).** Abbreviations: Anti-IL-6, anti-interleukine-6 receptor; RdRP, ARN polymerase gene; BMI, body mass index; COPD, chronic obstructive pulmonary disease; CT, cycle threshold; E, envelope gene; HCWs, health care workers; ICU, intensive care unit; GGO, ground-glass opacity; ORL, otorhinolaryngological; RT-PCR, real-time reverse transcription polymerase chain reaction; SARS-CoV-2, severe acute respiratory syndrome coronavirus 2; SD, standard derivation. ^1^ Defined by multiple sclerosis, Alzheimer’s disease, stroke, Parkinson disease. ^2^ Defined by community acquired pneumonia, emphysema, and obstructive sleep apneas. ^3^ Defined by panic attacks.

**Table 2:** Gustatory dysfunction in 229 COVID-19 patients with resolved or persistent olfactory dysfunction after infection with SARS-CoV-2, *Nord Franche-Comte* Hospital, *France*.

Variables	Proportion of Patients			*p*-Value
	Resolved Olfactory Dysfunction (*n* = 136) (59.4%)	Persistent Olfactory Dysfunction (*n* = 93) (40.6%)	Total (*n* = 229) (100%)	
Gustatory dysfunction rate % (no.)				
Total	81.6 (111)	87.1 (81)	83.4 (192)	0.356
Hypogeusia	21.3 (29)	30.1 (28)	24.9 (57)	0.176
Ageusia	60.3 (82)	57.0 (53)	59.0 (135)	0.717
*Duration*				
Recovery time of gustatory dysfunction, days (mean, extremes, SD)	26.0 [0–252] ± 34.0	25.8 [1–113] ± 29.3	25.9 [0–252] ± 33.1	0.985
*Persistent gustatory dysfunction*				
	*Over 111 patients*	*Over 81 patients*	*Over 192 patients*	
Gustatory dysfunction persistence rate % (no.)				
**Total**	**4.6 (5)**	**66.7 (54)**	**30.7 (59)**	**<0.001**
**Persistent hypogeusia**	**4.50 (5)**	**64.2 (52)**	**29.7 (57)**	**<0.001**
Persistent ageusia	0.0 (0)	2.50 (2)	1.04 (2)	0.164

**Bold: significant difference (*p* < 0.05).**

**Table 3:** Short version of questionnaire of olfactory disorders-negative statements of patient.

Short Version QOD-NS Items	Resolved OD Group (*n* = 136)	Persistent OD Group (*n* = 93)	Total (*n* = 229)	*p*-Value
Changes in my sense of smell isolate me socially.	2.18 ± 1.15	2.10 ± 1.15	2.14 ± 1.15	0.812
The problems with my sense of smell have a negative impact on my daily social activities.	2.03 ± 1.16	1.78 ± 1.15	1.93 ± 1.16	0.319
The problems with my sense of smell make me more irritable.	1.97 ± 1.14	1.75 ± 1.14	1.88 ± 1.14	0.116
Because of the problems with my sense of smell, I eat out less.	1.46 ± 1.36	1.37 ± 1.36	1.42 ± 1.36	0.091
Because of the problems with my sense of smell, I eat less than before (loss of appetite).	0.99 ± 1.13	0.94 ± 1.12	0.97 ± 1.13	0.567
Because of problems with my sense of smell, I must make more effort to relax.	1.92 ± 1.17	1.83 ± 1.16	1.88 ± 1.17	0.949
I am afraid I will never be able to get used to the problems with my sense of smell.	1.36 ± 1.22	0.97 ± 1.21	1.21 ± 1.21	0.102
Short version QOD-NS total score	12.0 ± 6.03	10.7 ± 5.89	11.0 ± 5.94	0.137

sQOD-NS is a seven-item patient-reported outcome questionnaire including social, eating, annoyance, and anxiety questions. Each item is rated on a scale of 0–3, with higher score reflecting better olfactory-specific quality of life. The total score ranges from 0 (sever impact on QoL) to 21 (no impact on QoL). sQOD-NS Short version of Questionnaire of Olfactory Disorders-Negative Statements.

## Data Availability

Data available on request due to privacy restrictions. The data presented in this case study are available on request from the corresponding author.
